# Gate-Tunable Plasmon-Induced Transparency Modulator Based on Stub-Resonator Waveguide with Epsilon-Near-Zero Materials

**DOI:** 10.1038/s41598-019-39047-y

**Published:** 2019-02-26

**Authors:** Long Tao, Aleksei Anopchenko, Sudip Gurung, Jinqiannan Zhang, Ho Wai Howard Lee

**Affiliations:** 1 Department of Physics, Baylor University, Waco, TX 76798 United States; 2 The Institute for Quantum Science and Engineering, Texas A&M University, College Station, TX 77843 United States

## Abstract

We demonstrate an electrically tunable ultracompact plasmonic modulator with large modulation strength (>10 dB) and a small footprint (~1 μm in length) via plasmon-induced transparency (PIT) configuration. The modulator based on a metal-oxide-semiconductor (MOS) slot waveguide structure consists of two stubs embedded on the same side of a bus waveguide forming a coupled system. Heavily n-doped indium tin oxide (ITO) is used as the semiconductor in the MOS waveguide. A large modulation strength is realized due to the formation of the epsilon-near-zero (ENZ) layer at the ITO-oxide interface at the wavelength of the modulated signal. Numerical simulation results reveal that such a significant modulation can be achieved with a small applied voltage of ~3V. This result shows promise in developing nanoscale modulators for next generation compact photonic/plasmonic integrated circuits.

## Introduction

As the dimensions of the transistors in integrated circuits continuously reduce to sub-10-nm, there is a significant mismatch between the sizes of silicon based photonic devices (micrometer range) and that of electronic elements (nanometer range)^1–3^. Interconnect is desired to eliminate this mismatch and build photonic-electronic hybrid circuit which takes advantage of the best of each element. Surface plasmon polariton structures have been suggested for integrated optical circuit since they can break the diffraction limit and confine light in nanoscale at optical frequencies^4^. Along with this idea, different nanoscale plasmonic devices which allow subwavelength confinement of the optical mode to integrate into highly compact optical circuits have been demonstrated^5,6^.

Among these plasmonic devices, electro-optical plasmonic modulators and modulators with epsilon-near-zero (ENZ) effect in transparent conducting oxides have been investigated extensively in the last few years^1,7–16^. Plasmonic modulators with modulation rates exceeding 100 GHz^17,18^, sub-micrometer modulators^19^ and even an atomic scale plasmonic switch^20^ have recently been demonstrated experimentally. However, their modulation properties need be further improved by achieving simultaneously a small footprint, high speed, and large modulation strength. In this work, we present a new plasmon-induced transparency (PIT) configuration tunable through the field effect in the ENZ conducting oxide. This electrically tunable PIT scheme can be used in many applications such as optical modulators, switches, data storage, precise optical measurements, sensors, etc. We demonstrate an efficient metal-oxide-semiconductor (MOS) based plasmonic modulator via a combined PIT and ENZ effect. Modulation strength larger than 10 dB are achieved with an applied bias of ~3V. This modulator with strong modulation strength, small footprint, fast switching, and low power consumption could find applications in next-generation ultra-compact and high speed integrated nanophotonic circuits and devices.

**Figure 1:**
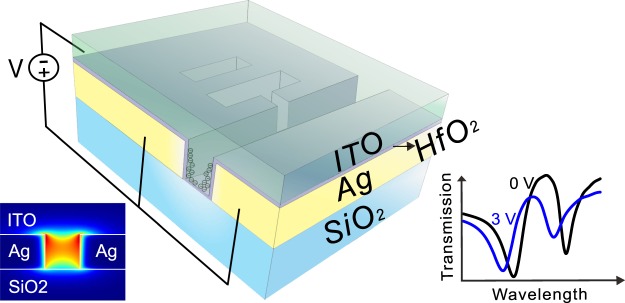
Schematic of ITO-filled plasmonic modulator showing a formation of accumulation layer at the ITO-HfO_2_ interface under external electrical bias. Left bottom inset shows the magnetic field distribution of the gap-plasmon mode inside the waveguide. Right bottom inset shows the output transmissions under 0V and 3V bias.

**Figure 2:**
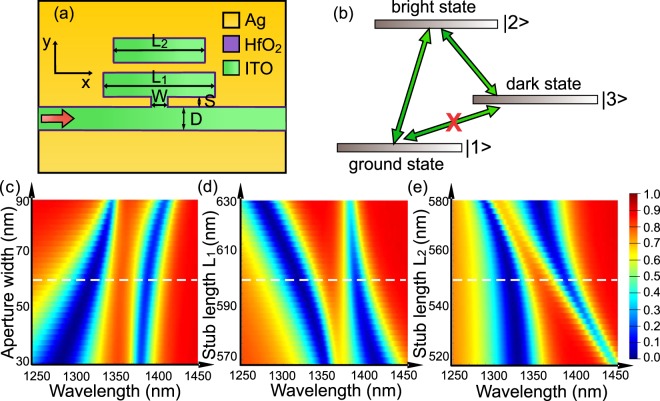
(**a**) Schematic of the modulator (top view). (**b**) Plasmonic analogue of electromagnetically induced transparency system. Variation of the simulated transmission with different (**c**) aperture widths, stub lengths (**d**) L_1_ and (**e**) L_2_. White dotted line shows the parameters we chose for the modulator design.

The plasmonic modulator consists of metal (Ag), oxide (HfO_2_), and semiconductor (ITO) layers as depicted in Fig. 1. The ITO-filled plasmonic waveguide supports gap-plasmonic mode (inset of Fig. 1) and reveals high active modulation of ~2.7 dB/μm via field-effect dynamics^1,21^. To enhance the field-effect modulation, a structure which has sharp resonance in transmission is desired. One of the most appealing approaches to achieve a sharp optical resonance is to make use of the PIT effect^22–26^, where a coupled-mode resonant structure is integrated with a plasmonic modulator (Fig. 2(a), top view)^27–31^. This configuration consists of two resonators (stub 1 and stub 2) and one bus waveguide, which are equally spaced along y-axis with separation S. Two stubs have the same width D as the bus waveguide, but different length *L*_1_ and *L*_2_ respectively. The stub 1 is connected to the bus waveguide by a small aperture with width W. The yellow, purple, and green shaded areas in the schematic represent silver, HfO_2_, and ITO separately. The thickness of the HfO_2_ layer which isolating the ITO and silver is 5 nm.

Plasmon-induced transparency is a classical analogue of the electromagnetically induced transparency (EIT)^32,33^ – a coherent phenomenon in atomic systems^22,34–40^. In an atomic three-level Λ (lambda) configuration shown in Fig. 2(b), the transitions between ground state $|1\rangle$ and bright state $|2\rangle$, as well as bright state $|2\rangle$ and dark state $|3\rangle$, are dipole allowed, while the transition between ground state $|1\rangle$ and dark state $|3\rangle$ is dipole forbidden. There are two coupling paths from $|1\rangle$ to $|2\rangle$: $|1\rangle \to |2\rangle \,$, and $|1\rangle \to |2\rangle \to |3\rangle \to |2\rangle$, the destructive interference between which induces a sharp and narrow spectral peak in transmission (inset of Figs 1 and 2(c–e)). Analogous to EIT, in our PIT configuration, the bus waveguide, stub resonator 1, and stub resonator 2 work as the ground state, bright state, and dark state, respectively^31,41,42^. The destructive interference occurs between light emerging from two coupled stub resonators. The PIT phenomenon results in a rapid change in absorption and positive variation in refractive index; thus light exhibits extremely low group velocity in this structure and could be used for delay lines in photonic circuits^22,43,44^. In this work, we use the EIT-like transmission of the PIT configuration for enhancing the modulation through the field-effect tunable ENZ.

The geometry is optimized in terms of the aperture width and the length of two stubs via parametric sweeps shown in Fig. 2(c–e). The color stands for transmission value varying from 0 to 1. We choose 60 nm for the aperture width to obtain the narrowest central peak. As for the stubs length, there is a trade-off between the narrow bandwidth of the transmission peak and highest transmission value. We choose 600 nm for the length of stub 1 and 550 nm for the length of stub 2 to retain narrow band transmission with reasonable transmission intensity for the modulator. As a result, the geometrical structure parameters in the modulation simulations are: *L*_1_ = 600 *nm*, *L*_2_ = 550 *nm*, *W* = 60 *nm*, *S* = 50 *nm*, *D* = 100 *nm* (Fig. 2a).

To determine the characteristics of this modulator, we first simulate the carrier concentrations and charge profiles (see Supporting Information Fig. S1 and Methods) of ITO corresponding for different gating voltages. The two-dimensional carrier concentration distributions in the MOS modulator under different applied voltages are calculated with background doping concentration ${N}_{BG}=1.0\times {10}^{19}\,c{m}^{-3}$. Positive voltages are applied on the two silver sides simultaneously, and ITO is connected to the negative terminal (Fig. 1). A non-ohmic contact boundary condition is used at the HfO_2_-Ag interface, so that the band bending and the electric field in the MOS structure are calculated as shown in Supporting Information Fig. S2. We used literature values for the electrical parameters of ITO layer (dielectric constant = 9.3, ${m}_{e}^{\ast }=0.28\,{m}_{e}$, ${E}_{bg}=2.5\,eV$, ${W}_{f}^{ITO}=4.75\,eV$, *μ*_*e*_ = $31\,c{m}^{2}/(V\cdot s)$)^45–48^. Here ${m}_{e}^{\ast }$ is the effective mass of electron, *m*_*e*_ is the mass of electron, *E*_*bg*_ is the band gap energy of ITO, ${W}_{f}^{ITO}$ is the work function of ITO, and *μ*_*e*_ is the electron mobility. The work function of silver is 4.26 eV^49^, and the dielectric constant of HfO_2_ is 25^48,50^.

**Figure 3:**
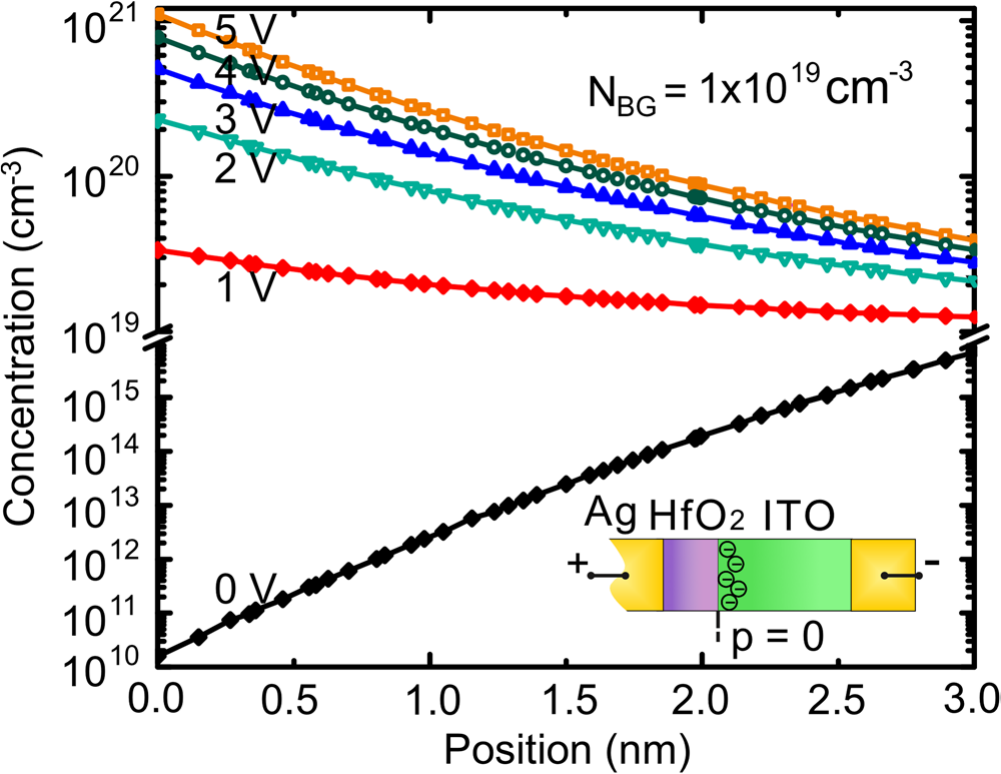
Carrier concentration distributions of ITO at the first 3 nm range from the ITO-HfO_2_ interface. Dots represent the data points obtained from electrical simulations. Inset: Schematic of the MOS structure. p = 0 stands for position exactly at the ITO-HfO_2_ interface.

Figure 3 shows the simulated carrier concentration distributions of ITO at the first 3 nm range from the ITO-HfO_2_ interface for different applied voltages. Without bias, a depletion layer appears at the ITO-HfO_2_ interface^45^, where the carrier concentration is lower than the background doping. However, the carrier density at the interface increases and turns to a flat-like distribution with 1V applied bias. The accumulation layer forms and carrier concentration at the ITO-HfO_2_ increases significantly with applied bias >1V. The maximum bias we can apply is limited by the breakdown voltage of HfO_2_. The bias of 5 V is still below the breakdown voltage of HfO_2_ reported in reference^51^.

With the obtained voltage-dependent carrier distribution, the change of the permittivity of ITO could be calculated using the Drude model:

1 $${\varepsilon }_{{\rm{ITO}}}={\varepsilon }_{\infty }-\frac{{\omega }_{p}^{2}}{{\omega }^{2}+i\omega {\rm{\Gamma }}},$$

where ${\omega }_{p}^{2}\,=N{e}^{2}/({m}_{e}^{\ast }\cdot {\varepsilon }_{0})$ and ${\rm{\Gamma }}=e/({m}_{e}^{\ast }\cdot {\mu }_{e})$. Here *ε*_0_ is the permittivity of free space, *e* is the elementary charge, *ε*_∞_ is the permittivity at infinite frequency equal to 3.6^52^, *ω*_*p*_ is the plasma frequency, Γ is the electron relaxation rate, and *N* is the carrier concentration.

**Figure 4:**
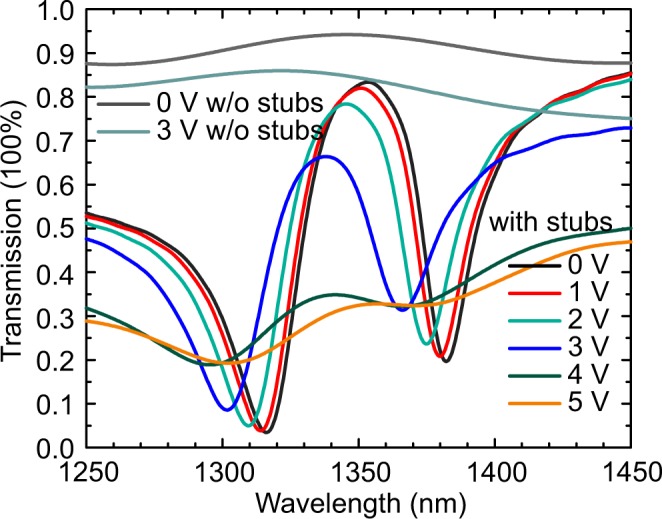
Transmission spectra of the waveguide modulator under different bias voltages. Transmission spectra of a straight waveguide (without stubs) are shown for comparison.

The optical simulation results for different applied voltages are shown in Fig. 4 and Supporting Information Fig. S3. For the structure without the coupled stubs and the aperture, the transmission is high and flat when the light transmits through the bus waveguide (gray curve in Fig. 4) at zero bias. The transmission decreases by 8–13% with the applied bias of 3V (gray blue curve in Fig. 4). The PIT configuration induces the resonances, which are mainly comprised of two transmission dips and one peak in the wavelength range of simulation. When the external voltage is applied, the resonances shift to shorter wavelength as seen in Fig. 4. The two dips located at wavelength of 1316 nm and 1382 nm at 0V (black curve in Fig. 4) shift for 15 nm and 16 nm respectively at 3V (blue curve in Fig. 4), and the transmission is attenuated. The transmission decreases significantly with further increase of voltages due to a sharp increase in the loss of the waveguide (see Fig. S4(a) in the Supporting Information). Because of this, we mainly focus on the modulation with a small voltage (3V), which can keep the sharp resonances, meanwhile the waveguide loss is still below 1 dB/μm.

**Figure 5:**
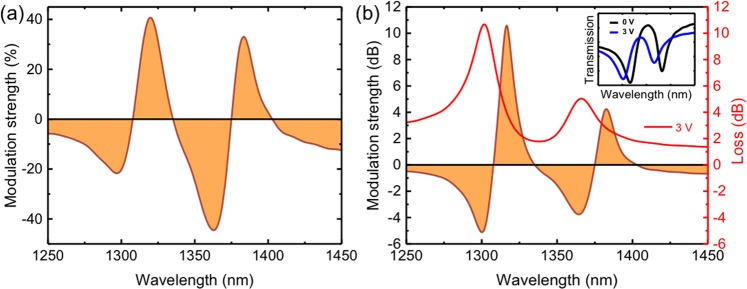
Modulation strength of the plasmonic modulator (**a**) in percentage scale and (**b**) in dB scale with loss at applied bias of 3V. Inset: Transmission spectra with applied bias of 0V and 3V.

The corresponding modulation strength from the results of Fig. 4 is shown in Fig. 5(a,b), which show the modulation strength with 3V bias in percentage scale and dB scale respectively. The inset in Fig. 5(b) shows the transmissions particularly at 0V and 3V. It can be seen from the figure that amplitude modulation with more than 40% (or >10 dB) near the PIT resonance frequency is achieved. Figure 5(b) also shows the total PIT modulator loss obtained from the transmission spectra (Fig. 4) under the applied bias of 3V. At the wavelength of the maximum modulation strength, the off-state (the low loss state) loss is <4 dB. It is noteworthy that larger modulation strength (~14 dB) and lower losses <3 dB are obtained for shorter separation distance between the stub resonator and the bus waveguide, namely S = 45 nm (see Fig. S6 in the Supporting Information). The small device footprint of 1.4 µm, extinction ratio >10 dB, and low waveguide loss and total loss <3 dB (in the off-state) make the PIT modulator comparable with state-of-the-art traditional noble-metal-based and novel alternative material plasmonic modulators, which have extinction ratio vs. propagation losses (figure of merit) values <10 ^7,13,53^. The modulation strength is more than four times higher than the demonstrated TCO PlasMOStor^1^ or conducting oxide modulator^9^ using the similar conducting oxide field-effect dynamic.

It should be noticed that the modulation strength could be further enhanced by changing the geometries of the waveguide or the coupled resonators. Since the change of the carrier concentration only occurs near the ITO-HfO_2_ interface, reducing the width of the two stubs could enhance the interactions between the resonant optical mode and the accumulated electrons. For instance, a modified geometry with shorter width (90 nm) of the bus waveguide and two stubs (see Fig. S5 in the Supporting Information) shows higher modulation strength than one in Fig. 5b, indicating that the modulation strength of the modulator could be further enhanced by geometry optimization which is beyond the scope of this paper. Our simulations of the mode properties of the plasmonic waveguide (see Supporting Information Fig. S4) shows the frequency dependence of the loss of the bus waveguide under various bias. The loss is 0.3 dB/μm at 0V and less than 0.9 dB/μm at 3V in the full wavelength range of simulation, indicating the low insertion loss of the device as only 1–2 μm device length is required in our structure. It is important to mention that the designed geometric dimensions of the plasmonic waveguide and stub resonators could be realized by the standard electron beam lithography and lift-off/etching processes or advanced helium focused ion beam milling technique to fabricate features <100 nm with a fabrication uncertainty of ~5 nm^1,54^. This fabrication uncertainty will not deteriorate the plasmonic modulator performance. For example, varying the separation distance *S* between the side-coupled stub and the bus waveguide by 5 nm in a range of 40–60 nm does not affect the modulator performance significantly (see Supporting Information Figs S6 and S7) indicating the robustness of the design.

**Figure 6:**
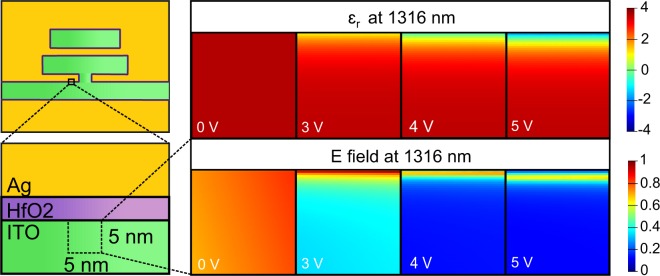
Real part of permittivity and electric field profile in ITO at the first 5 nm range from the ITO-HfO_2_ interface corresponding to different biases at the wavelength of 1316 nm.

To gain further insight on the origin of the enhanced modulation, we investigate the field confinement along the waveguide corresponding to the field-effect induced permittivity change. Figure 6 shows the real part of permittivity and the electric field profile at the first 5 nm from the ITO-HfO_2_ interface for different voltages at the wavelength of 1316 nm (the left resonance dip at 0V in Fig. 4). Without applied bias, the ITO behaves like dielectric and the electric field is uniform at the first few nanometers in the interface. Under 3V applied bias, the permittivity at the interface nears zero due to the formation of the accumulation layer, i.e. the ENZ condition is met^48^. Consequently, the electric field confine strongly in the accumulation layer of ITO at the ENZ condition (bottom, Fig. 6). ITO interface shows metallic and ENZ properties (*ε*_*r*_ < 0) when the voltage is above 3V. Due to the coupling of the metallic and ENZ region, the field profile of the gap-plasmonic waveguide mode significantly modified, resulting in a large change of the resonance transmission (Fig. 5). Therefore, the modulation properties relied strongly on the formation and the shift of the ENZ active layer near the ITO-HfO_2_ interface under external bias. Note that the reported modulation properties have not been fully optimized. Rigorous optimization of the separation between the bus waveguide and the stubs, and the dimensions of the stubs, could potentially enhance the modulator performance. In addition, the accumulation/depletion layer depends strongly on the material properties such as the Fermi level, the energy bandgap, the electron affinity, and the dielectric constant of the insulator. Proper selection and optimization of the metal/insulator/semiconductor structure will also increase the electron density at the interface and the Debye length, thus enhancing the modulator performance.

We further evaluate the performance of the PIT modulator by determining the power consumption and switching time of the device. Our numerical results show that a device of 1.4 μm length is sufficient to induce more than 10 dB modulation strengths. Based on the simulated carrier concentration distribution shown in Fig. 3, we obtain the capacitance and change of charge for different applied voltages (see Supporting Information Fig. S8). From our calculation, the capacitance varies from 16 fF/μm^2^ to 32 fF/μm^2^, e.g. the capacitance at 3V is 30.7 fF/μm^2^. Comparing to the work by Lee *et al*.^1^ in which Al_2_O_3_ is used as the insulating oxide, the capacitance of our designed modulator is higher, due to the higher dielectric constant of HfO_2_ than Al_2_O_3_. Since there are two resonators coupled to the bus waveguide, all the Ag-HfO_2_ boundaries need to be counted for the total length. Assuming the thickness of the modulator of 200 nm^1^, the total area of Ag-HfO_2_ interface is 1.336 μm^2^ and the corresponding total capacitance is 41 fF. Then we estimate the power consumption as 1/4 *CV*^2,1,55^, where *C* is capacitance and *V* is the applied voltage. The estimated power consumption of this plasmonic modulator is 90.2 *fJ*/*bit*, which is comparable with the state-of-the-art values^1,12,17,18,56^. Because of the small footprint and large modulation strength, this power consumption is commendable and could be further reduced by decreasing the capacitance through material optimization, etc. In addition, we estimate the operation speed of this modulator assuming that it is an RC circuit limited as 1/(2*πRC*). Neglecting other parasitic contributions and assuming a driver impedance of 50 Ω^15,57^, our modulator enables modulation speed up to 78 GHz. Further reducing the dimensions of the modulator could enable a higher switching speed. The ultra-compact, large modulation strength and high operation speed of this ENZ-tunable PIT modulator can find applications in on-chip optical communication, electro-optic switches, etc. In addition, this plasmonic modulator can be integrated with Si based photonics to reduce the propagation loss induced by the plasmonic waveguide^56,58^.

In conclusion, we reported an efficient and ultracompact conducting oxide plasmonic modulator with PIT configuration. The modulator has large modulation strength >10 dB, total device loss in the off-state <3 dB, low power consumption of 90 fJ/bit, and compact dimension of ~1 μm. These plasmonic modulators can be useful for integrated nanoscale switching devices for next generation photonic/plasmonics or plasmonics/electronics integration.

## Methods

Numerical simulations of the carrier concentration distributions, electric field and conduction band bending in the MOS modulator under different applied voltages were carried out using the DEVICE Solutions software from Lumerical Solutions, Inc. The device simulator uses the finite element method to self-consistently solve the Poisson and drift-diffusion equations. The ohmic contacts at the metal and ITO outer interfaces are set as the boundary conditions (see the inset of Figs 3 and S2). A self-adaptive mesh generation algorithm is used with maximum refinement steps of 20,000 and minimum mesh size of 5 × 10^−4^ nm in our electrical simulations. The ITO band structure parameters used in the simulations are: dielectric constant = 9.3, ${m}_{e}^{\ast }=0.28\,{m}_{e}$, ${E}_{bg}=2.5\,eV$, ${W}_{f}^{ITO}=4.75\,eV$, ${\mu }_{e}=31\,c{m}^{2}/(V\cdot s)$ ^45–48^. Here ${m}_{e}^{\ast }$ is the effective mass of electron, *m*_*e*_ is the mass of electron, *E*_*bg*_ is the band gap energy of ITO, ${W}_{f}^{ITO}$ is the work function of ITO, and *μ*_*e*_ is the electron mobility. The work function of silver is 4.26 eV^49^, and the dielectric constant of HfO_2_ is 25^48,50^. The calculated charge profiles and permittivity distribution were then imported directly for full-wave electromagnetic simulation (FDTD Solutions by Lumerical, Inc., which is compatible with DEVICE). The permittivities of Ag and HfO_2_ are from ref.^40^ and ref.^59^. The mesh size of 0.1 nm is used in all optical simulations. The loss and effective refractive index of the bus waveguide were calculated using the MODE Solutions software from Lumerical Solutions, Inc.

## Supplementary information

Supporting Information_ Gate-Tunable Plasmon-Induced Transparency Modulator Based on Stub-Resonator Waveguide with Epsilon-Near-Zero Materials

## Data Availability

The datasets generated during and/or analyzed during the current study are available from the corresponding author on request.
