# Supplementary material for: Gate-Tunable Plasmon-Induced Transparency Modulator Based on Stub-Resonator Waveguide with Epsilon-Near-Zero Materials
Source: Sci Rep. 2019 Feb 26;9:2789. doi: 10.1038/s41598-019-39047-y (PMC6391484; doi:10.1038/s41598-019-39047-y)
Supplement: Supplementary file 1 — Supporting Information_ Gate-Tunable Plasmon-Induced Transparency Modulator Based on Stub-Resonator Waveguide with Epsilon-Near-Zero Materials [file 41598_2019_39047_MOESM1_ESM.pdf]

Supporting Information

Gate-Tunable Plasmon-Induced Transparency Modulator Based on Stub-Resonator Waveguide with Epsilon-Near-Zero Materials

Long Tao[1], Aleksei Anopchenko[1], Sudip Gurung[1], Jinqiannan Zhang[1], and Ho Wai Howard Lee [1,2 †]

[1]Department of Physics, Baylor University, Waco, TX 76798, United States

[2]The Institute for Quantum Science and Engineering, Texas A&M University, College Station, TX 77843, United States

[†]Corresponding author e-mail: Howard_Lee@Baylor.edu

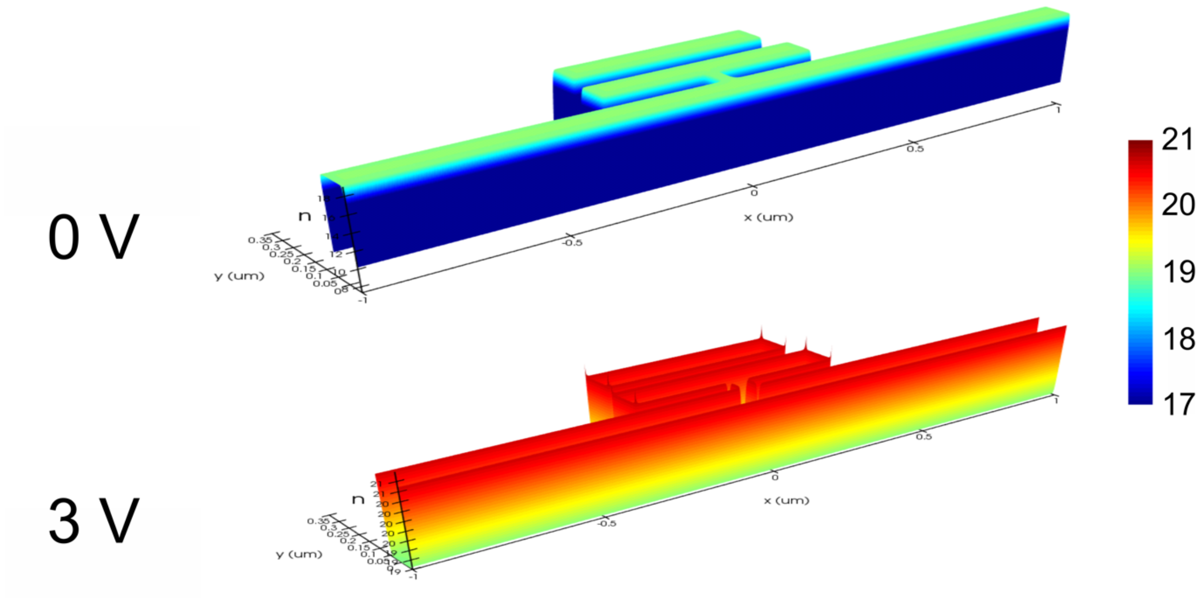

Fig. S1. Charge profiles of waveguide modulator under 0 V (depletion) and 3 V (accumulation), where x, y axes are Cartesian coordinates corresponding to Fig. 2(a), vertical axis stands for the carrier concentration in log scale.

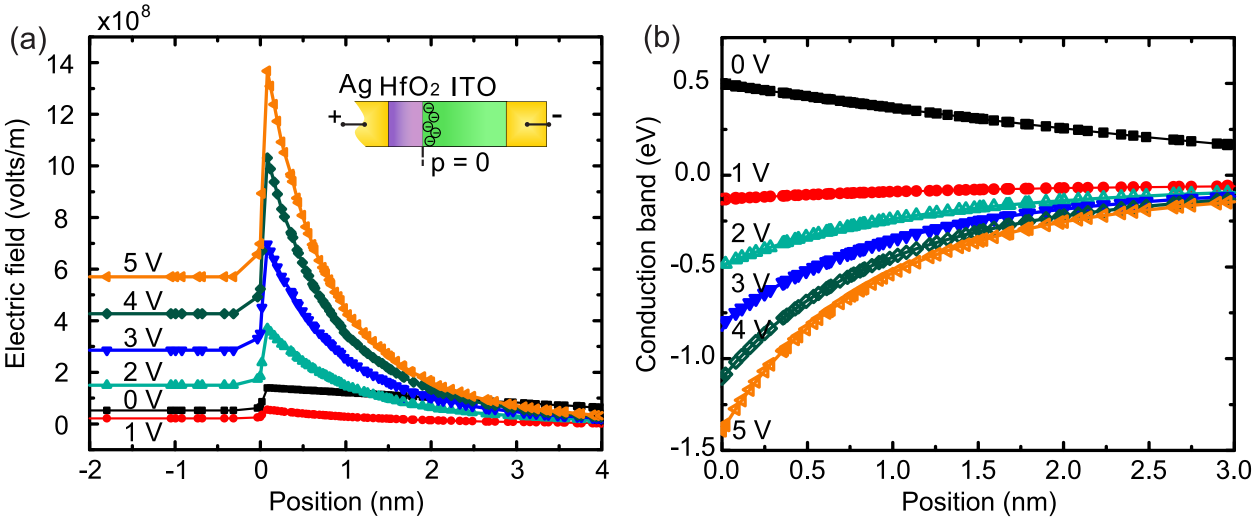

Fig. S2. Electric field (a) and conduction band bending (b) near the ITO-HfO_2 interface. Dots represent the data points obtained from electrical simulations. Inset: Schematic of the MOS structure. p = 0 stands for position exactly at the ITO-HfO_2 interface.

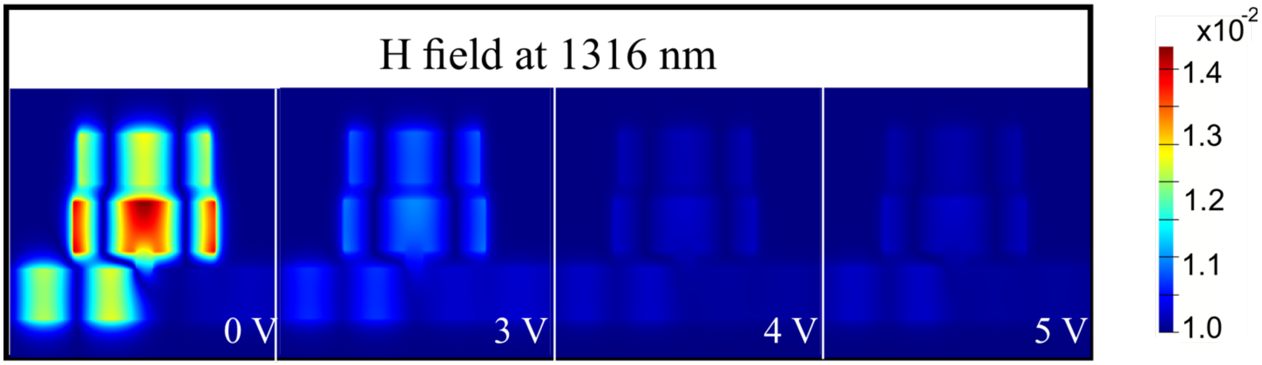

Fig. S3. Magnetic field profile in the waveguide modulator (top view) at 1316 nm with different biases. Strong field localization occurs at 0 V.

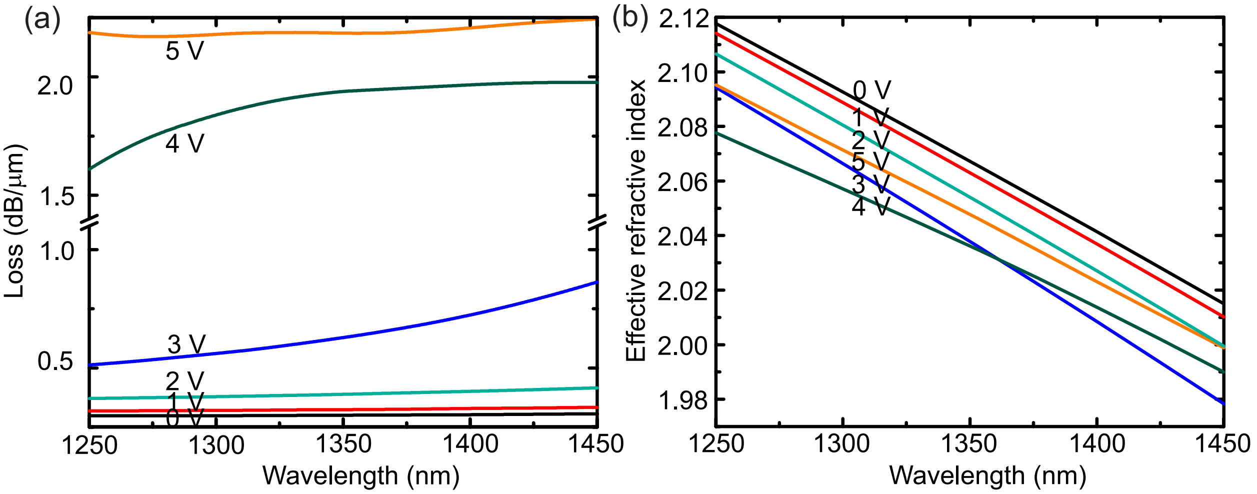

Fig. S4. Loss (a) and effective refractive index (b) of the bus waveguide under different biases. The waveguide loss increases significantly with the applied bias higher than 3 V.

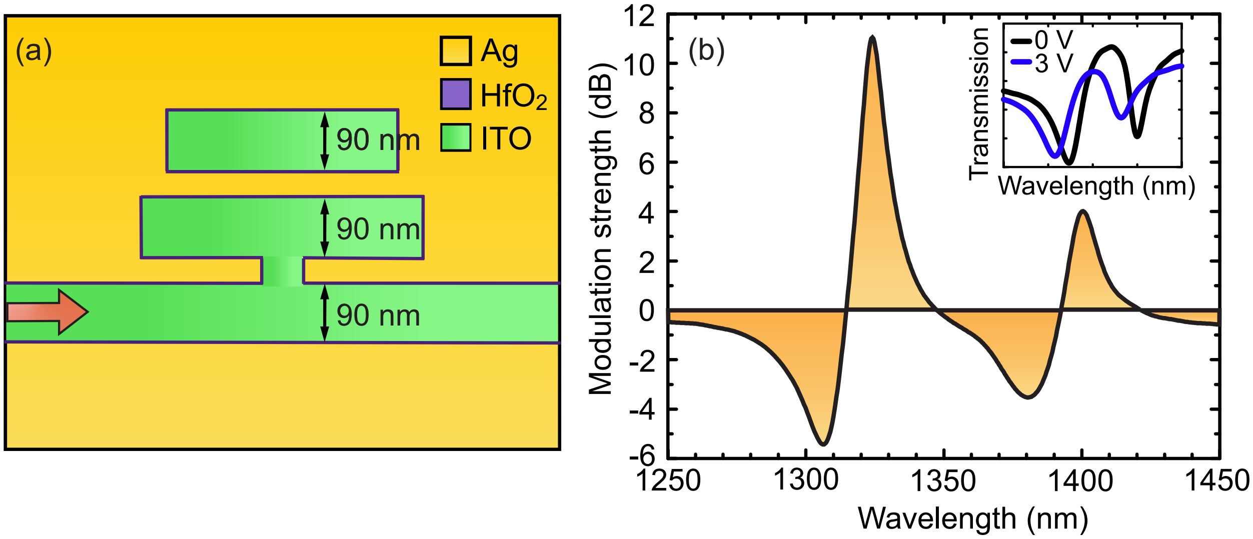

Fig. S5. (a) Schematic of the revised waveguide modulator with 90 nm width for bus waveguide and two stubs. (b) The corresponding modulation strength. Inset: transmission spectra at 0 V and 3 V applied voltages.

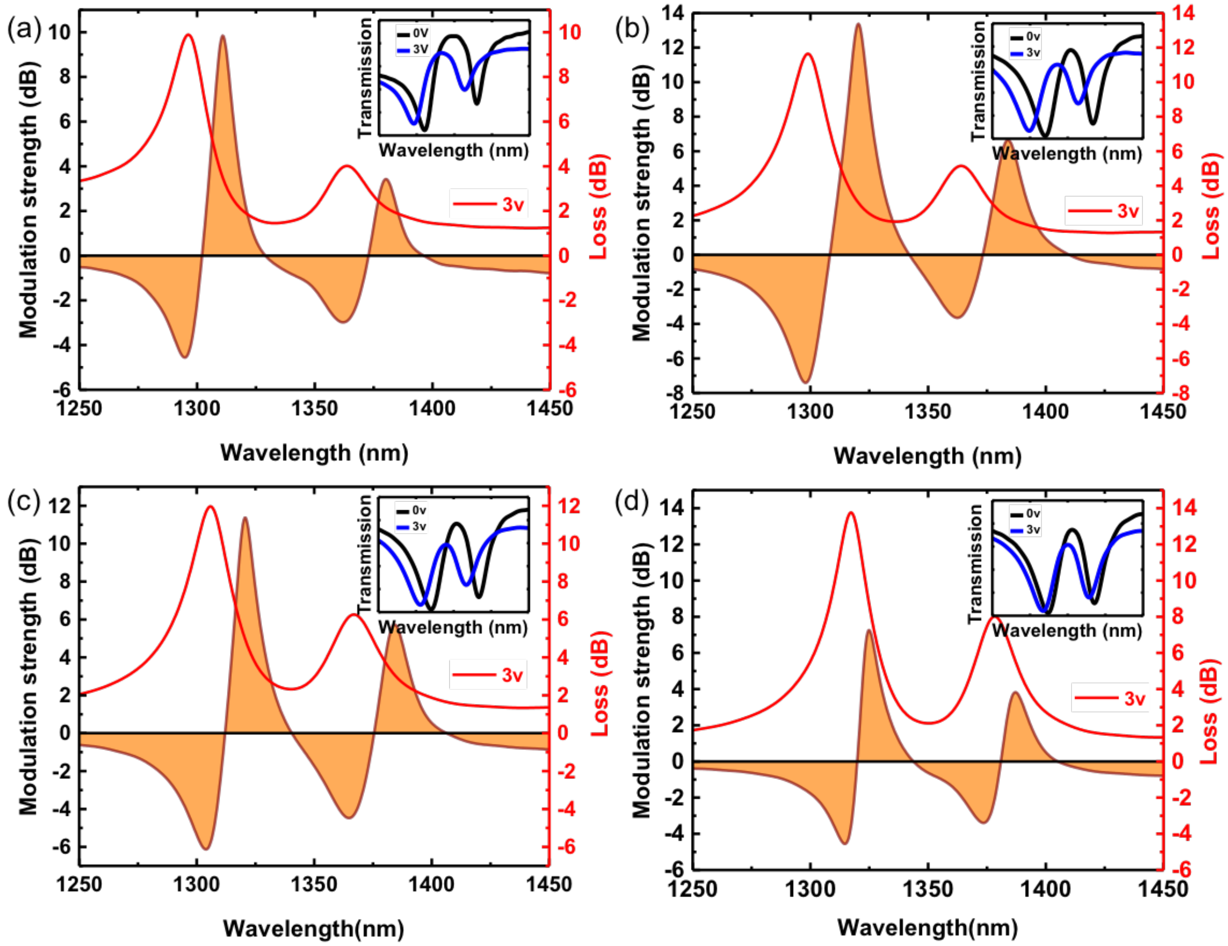

Fig. S6. Modulation strength of the plasmonic modulator with (a) S = 40 nm, (b) S = 45 nm, (c) S = 55 nm and (d) S = 60 nm. Other structural parameters of the modulator remain the same, namely $L_1 = 600\ nm$, $L_2 = 550\ nm$, $W = 60\ nm$, $D = 100\ nm$. Inset: transmission spectra at applied bias of 0 V and 3 V.

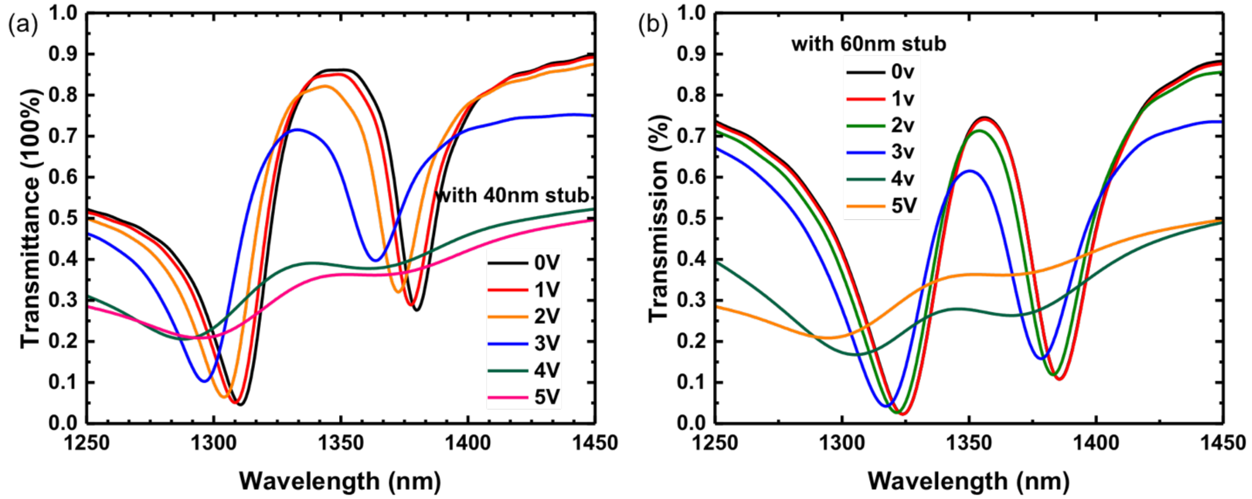

Fig. S7. Transmission spectra of the plasmonic modulator with (a) $S = 40$ nm and (b) $S = 60$ nm under different bias voltages. Other geometrical structure parameters of the modulator remain the same: $L_1 = 600\ nm$, $L_2 = 550\ nm$, $W = 60\ nm$, and $D = 100\ nm$.

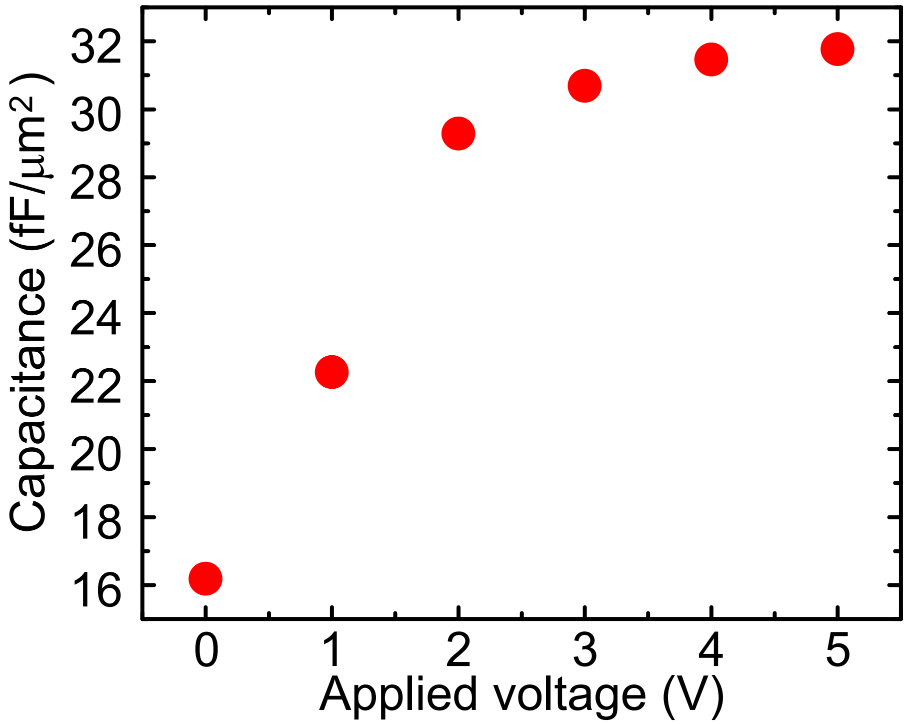

Fig. S8. Capacitance of the waveguide modulator as a function of the applied bias.
